# Exposure to Secondhand Smoke Outside of a Bar and a Restaurant and Tobacco Exposure Biomarkers in Nonsmokers

**DOI:** 10.1289/ehp.1104413

**Published:** 2012-04-06

**Authors:** Gideon St.Helen, J. Thomas Bernert, Daniel B. Hall, Connie S. Sosnoff, Yang Xia, John R. Balmes, John E. Vena, Jia-Sheng Wang, Nina T. Holland, Luke P. Naeher

**Affiliations:** 1 Department of Environmental Health Science, College of Public Health, University of Georgia, Athens, Georgia, USA; 2 Division of Laboratory Sciences, National Center for Environmental Health, Centers for Disease Control and Prevention, Atlanta, Georgia, USA; 3 Department of Statistics, University of Georgia, Athens, Georgia, USA; 4 Division of Occupational and Environmental Medicine, School of Medicine, University of California-San Francisco, San Francisco, California, USA; 5 Department of Epidemiology and Biostatistics, College of Public Health, University of Georgia, Athens, Georgia, USA; 6 Division of Environmental Health Sciences, School of Public Health, University of California–Berkeley, Berkeley, California, USA

**Keywords:** NNAL, outdoor secondhand smoke, salivary cotinine, smoking bans, tobacco nitrosamines

## Abstract

Background: With an increase in indoor smoking bans, many smokers smoke outside establishments and near their entrances, which has become a public health concern.

Objectives: We characterized the exposure of nonsmokers to secondhand smoke (SHS) outside a restaurant and bar in Athens, Georgia, where indoor smoking is banned, using salivary cotinine and urinary 4-(methylnitrosamino)-1-(3-pyridyl)-1-butanol (NNAL).

Methods: In a crossover study, we assigned 28 participants to outdoor patios of a restaurant and a bar and an open-air site with no smokers on three weekend days; participants visited each site once and stayed for 3 hr. We collected saliva and urine samples immediately before and after the visits (postexposure) and on the following morning and analyzed samples for cotinine and total NNAL, respectively. Regression models were fitted and changes in biomarkers were contrasted between locations.

Results: Postexposure and preexposure geometric mean salivary cotinine concentrations differed by 0.115 ng/mL [95% confidence interval (CI): 0.105, 0.126)] and by 0.030 ng/mL (95% CI: 0.028, 0.031) for bar and restaurant visits, respectively. There were no significant post- and preexposure differences in cotinine levels after control site visits, and changes after bar and restaurant site visits were significantly different from changes after control site visits (*p* < 0.001). Results comparing next-day and preexposure salivary cotinine levels were similar. Next-day creatinine-corrected urinary NNAL concentrations also were higher than preexposure levels following bar and restaurant visits [1.858 pg/mg creatinine higher (95% CI: 0.897, 3.758) and 0.615 pg/mg creatinine higher (95% CI: 0.210, 1.761), respectively], and were significantly different from changes after the control visits (*p* = 0.005).

Conclusion: Salivary cotinine and urinary NNAL increased significantly in nonsmokers after outdoor SHS exposure. Our findings indicate that such exposures may increase risks of health effects associated with tobacco carcinogens.

Secondhand smoke (SHS) is a combination of smoke emitted from a burning tobacco product and the smoke exhaled by the smoker [Department of Health and Human Services (DHHS) 1986]. Scientific evidence continues to show that SHS exposure is causally associated with lung cancer among never-smokers or among nonsmokers (Vineis et al. 2007). Secondhand smoke increases the risk of cardiovascular disease by approximately 30% (Barnoya and Glantz 2005) and the risk of respiratory diseases (Flouris et al. 2009).

The increasing and overwhelming body of evidence that demonstrates elevated disease risk among nonsmokers exposed to SHS has led to the passage of smoking bans in workplaces and public places, including restaurants and bars. Smoke-free air laws have been very effective in reducing exposures to constituents of SHS (Bondy et al. 2009) as well as decreasing SHS-related diseases (Herman and Walsh 2010). In 2005, the state of Georgia passed a state-wide smoking ban in restaurants and bars that serve or employ minors (Georgia Smoke-free Air Act 2005). Athens-Clarke County in Georgia further implemented an ordinance in 2005 prohibiting smoking in all restaurants and bars but not in all workplaces (Athens-Clarke County, Georgia, Code of Ordinances, Section 4.3, 2005).

Despite large positive effects of smokefree air laws on public health, indoor smoking bans seem to result in increased smoking outside establishments, in outdoor seating areas, or near the entrances of such establishments. The magnitude of outdoor SHS exposure and associated health risks are relatively unknown as very few studies have characterized outdoor SHS exposure [California Air Resources Board (CARB) 2005; Kaufman et al. 2010; Klepeis et al. 2007; St.Helen et al. 2011)]. Systemic human exposure estimates from environmental markers reported in these studies are subject to factors such as proximity to smokers and meteorological conditions and may be highly imprecise (Benowitz 1999). We also previously reported the first biomonitoring of nonsmokers exposed to outdoor SHS using salivary cotinine in a pilot study (Hall et al. 2009). Cotinine is the primary proximate metabolite of nicotine.

Secondhand smoke contains carcinogenic tobacco-specific nitrosamines (TSNA) such as 4-(methylnitrosamino)-1-(3-pyridyl)-1-butanone (NNK) (Hoffmann and Hecht 1990). Uptake of NNK by nonsmokers exposed to SHS has been identified as a biochemical link between SHS exposure and lung cancer risk (DHHS 2006). NNK is mainly metabolized to 4-(methylnitrosamino)-​1-(3-pyridyl)-​1-butanol (NNAL) and the glucuronide conjugate (NNAL-Gluc) (Hecht 1996). Although cotinine is appropriate as a biomarker of SHS exposure it may not always be an accurate measure of exposure to some toxicants in tobacco smoke such as NNK (Benowitz et al. 2010), and thus underestimate SHS health risks. No studies have reported NNAL levels in nonsmokers who were exposed to outdoor SHS.

In this study, we used salivary cotinine to characterize the exposure of nonsmokers to SHS in outdoor seating areas and outside a restaurant and a bar in Athens, Georgia, where only indoor smoking is banned, and urinary NNAL to characterize their uptake of TSNA.

## Materials and Methods

*Study location.* We conducted this crossover study during three weekends in August and September 2010 in the college town of Athens, Georgia. Three locations were selected—outdoor seating or standing areas of a bar and a family restaurant where indoor smoking was banned, but both establishments had no restrictions on outdoor smoking, and an open air seating area outside the Environmental Health Science (EHS) building at the University of Georgia (UGA) with no smokers present. Descriptions of the study sites are presented in Table 1. The bar was selected based on previous data showing relatively high SHS outside this site (Hall et al. 2009; St.Helen et al. 2011). Although we previously measured lower SHS at family restaurants in Athens, Georgia, (Hall et al. 2009; St.Helen et al. 2011), we included a restaurant site in the present study because restaurants may serve as potential SHS sources to children and individuals who do not visit bars.

**Table 1:** Description of study sites.

Site	Bar	Restaurant	Control
Description		Bar with outdoor patio on second floor; partially enclosed by two walls of adjacent buildings, open at one end with no roof		Family restaurant with large open-air patio		Open-air seating
Location		Downtown Athens, GA; 5 min from UGA’s EHS building		Athens west; 10 min from EHS		Outside EHS building
No. of tables		6		17		5
Approximate outdoor area (m2)		176		549		NA
Cigarette Counta						
Mean ± SD		144.5 ± 39.9		33.5 ± 28.0		0
Minimum–Maximum		86–202		12–86		0
NA, not applicable. aCigarette count computed from 3-hr sums of 10-min cigarette count.						

*Participant recruitment and selection.* Participants were UGA college students. We administered a questionnaire, designed by our research team, to determine the elibility of potential participants. Questions included current and past smoking status and current SHS exposure at home, work, or elsewhere. Eligible participants were healthy males and females 21–40 years of age who did not use tobacco or nicotine in any form, females were not or could not be pregnant, and enrollment was directed to a target sample size of 24 participants. Respondents who met the eligibility requirements attended personal information sessions in which the study and protocol were discussed and concerns or questions were addressed. The study was designed as a crossover study in which participants visited each site once during each of the 3 weeks of the study (i.e, once each at the bar and restaurant sites and at the control site). The order of site visits was based on a replicated Latin square (Fleiss 1986) [see Supplemental Material, Table 1 (http://dx.doi.org/10.1289/ehp.1104413)]. Twenty-eight participants were enrolled in the study; each gave written informed consent before participating. The institutional review boards at UGA and the Centers for Disease Control and Prevention (CDC) approved the study.

*Site visits.* We asked participants to avoid all SHS as much as possible 3 days prior to each study weekend. Participants arrived at the EHS building about 1 hr before site visits. We collected preexposure saliva and urine samples from the participants. They were transported to the restaurant and bar sites on a nonsmoking EHS van. On study days, the participants visited the restaurant site and the control site at 1800–2100 hours and the bar site at 2300–0200 hours. Participants remained at each study site for the full 3 hr except for necessary bathroom breaks; they were encouraged to stand or sit in close proximity to smokers, which ranged from about 0.5 m to 5 m at any given time. Participants ate dinner while they were at the restaurant and control site and ate dinner before visits to the bar site. One participant at each location [a lab technician or graduate student in the EHS Air Quality Lab (AQL) who was familiar with the study protocol] was assigned to count and record the number of cigarettes that were lit during every 10-min period of the 3-hr visit. After the 3-hr visit, participants at the control site returned to the EHS building, and those at the restaurant and bar sites were transported on the EHS van. We collected postexposure saliva and urine samples within 30 min of participants leaving the study sites to assess the exposure of the participants to SHS for the 48-hr period before the site visits.

*Biological sample collection.* Participants provided saliva and urine samples immediately before and after site visits (referred to as preexposure and postexposure samples, respectively) to AQL staff at the EHS building. In addition, participants collected saliva and urine (first-morning void) at their homes on the next day (referred to as next-day samples), that were kept frozen until delivery by the participants to EHS on the Monday following each study weekend. Salivettes (Sarstedt, Newton, NC) were used to collect saliva samples. Participants were provided labeled urine sampling cups and salivettes for next-day sampling after the postexposure samples were collected. Samples were stored in a –80^o^C freezer at EHS until they were shipped on dry ice to the CDC (Atlanta, GA) for analyses of salivary cotinine and urinary NNAL and creatinine 6 weeks after the end of the study.

*Biomarker analysis.* Salivary cotinine was measured by high-performance liquid chromatography–atmospheric-pressure chemical ionization–tandem mass spectrometry (LC APCI MS/MS) using a method described elsewhere (Bernert et al. 2000); the limit of detection (LOD) was 15 pg/mL). We measured total NNAL (free plus conjugated) with high-performance liquid chromatography coupled with electrospray-ionization–tandem mass spectrometry using a method that has been described in Xia and Bernert (2010; LOD 0.6 pg/mL). We measured creatinine in urine by a commercially available colorimetric enzymatic method (Creatinine plus, version 2; Roche Diagnostics Corp, Indianapolis, IN) implemented on a Roche/Hitachi Modula P Chemistry Analyzer (Roche Diagnostics).

*Statistical analysis.* Because salivary cotinine and urinary NNAL values were not normally distributed, we computed geometric mean concentrations and used log-transformed values in regression analyses. Differences in geometric means and 95% confidence intervals (CIs) of preexposure, postexposure, and next-day biomarker levels were computed and used to represent changes in biomarker data after visits to each of the three sites. When biomarker levels were below their respective LODs, we used the reported concentrations obtained from the assays for cotinine and NNAL cited above. Our analyses were performed for both uncorrected and creatinine-corrected urinary NNAL.

We used regression analysis to contrast the changes in log-transformed biomarker levels from the preexposure samples to the postexposure and next-day samples according to location. For response *y*_ijk_ measured on the *k*th participant on the *j*th measurement occasion (day) under the *i*th exposure location, Equation 1 was assumed. In this study design, participants were assigned only 1 of 2 weekend days (Friday or Saturday), thus, day was nested in week (*w*_(j)l_):

*y*_ijk_ = μ_i_ + *w*_(j)l_ + *s*_k_ + *e*_ijk_, [1]

where μ_i_ represents the mean response for the *i*th exposure location, and *w*_(j)l_ and *s*_k_ are mean zero, constant variance, normal random effects for day nested in weeks and participants, respectively. To include nondetectable concentrations of NNAL (zero concentration) in regression analyses, we added a small number (10^–7^) to all NNAL values before log transformation. We conducted *F*-tests of no overall effect of exposure location as well as pair-wise contrasts between the control, restaurant, and bar locations, respectively, adjusted by Tukey’s method for multiple comparisons. To assess whether there was a carryover effect in biomarker response after SHS exposure from week to week, we conducted a repeated measures analysis on preexposure biomarker levels and contrasts were made across the 3 study weeks. Further, to examine the effect of number of cigarettes smoked at the different locations on changes in biomarker concentrations, we replaced the location variable shown in equation 1 with smoker count (total number of cigarettes counted over the 3-hr study period) in a second set of models.

Spearman rank correlation coefficients between changes in cotinine, NNAL, and creatinine-corrected NNAL were computed. Although cotinine is generally used as a biomarker of tobacco smoke exposure, it has not been shown to have adverse health effects. On the other hand, NNAL has comparable carcinogenic potency as its parent compound, NNK. Thus, it is important to determine the association between cotinine and NNAL after outdoor SHS exposure. Also, the ratio of NNAL to cotinine is of potential interest as a biomarker to discriminate passive from active smokers (Goniewicz et al. 2011). Thus, we examined differences in this ratio across locations using Kruskal–Wallis test [see Supplemental Material, Table 2 (http://dx.doi.org/10.1289/ehp.1104413)].

**Table 2:** Salivary cotinine and urinary NNAL in nonsmokers after visits to outdoor locations.

Analyte	Location	Variable	Preexposure	Postexposure	Next day
Cotinine (ng/mL)		Control		n		26		26		26
				Range		0.019–0.480		0.021–0.434		0.024–0.359
				GM		0.049		0.044		0.053
				95% CIa		0.037, 0.063		0.034, 0.058		0.041, 0.070
		Restaurant		n		27		23		25
				Range		0.011–0.165		0.036–0.188		0.029–0.181
				GM		0.046		0.075		0.069
				95% CI		0.036, 0.058		0.064, 0.089		0.058, 0.082
		Bar		n		27		25		26
				Range		0.015–0.356		0.094–0.407		0.035–0.444
				GM		0.045		0.161		0.165
				95% CI		0.035, 0.059		0.140, 0.184		0.136, 0.200
NNAL (pg/mL)		Control		n		27		27		26
				Range		0 –11.3		0–6.900		0–6.300
				GM		0.033		0.050		0.038
				95% CI		0.005, 0.203		0.008, 0.302		0.005, 0.263
C-C NNAL (pg/mg creatinine)				Range		0–4.061		0–2.724		0–3.099
				GM		0.038		0.057		0.030
				95% CI		0.007, 0.191		0.012, 0.285		0.005, 0.198
NNAL (pg/mL)		Restaurant		n		27		27		27
				Range		0–10.900		0–2.100		0–7.300
				GM		0.041		0.008		0.774
				95% CI		0.007, 0.239		0.001, 0.047		0.268, 2.234
C-C NNAL (pg/mg creatinine)				Range		0–15.875		0–1.921		0–10.501
				GM		0.056		0.013		0.671
				95% CI		0.011, 0.274		0.002, 0.069		0.221, 2.035
NNAL (pg/mL)		Bar		n		27		27		27
				Range		0–10.200		0–3.600		0–10.400
				GM		0.037		0.109		2.407
				95% CI		0.007, 0.206		0.023, 0.503		1.068, 5.425
C-C NNAL (pg/mg creatinine)				Range		0–95.500		0–3.749		0–6.113
				GM		0.039		0.182		1.898
				95% CI		0.007, 0.229		0.044, 0.755		0.904, 3.986
Abbreviations: C-C, creatinine corrected; GM, geometric mean; n, number of participants. a95% CI of geometric means.										

Finally, because nondetectable concentrations and below LODs made up 28.9% and 14.9%, respectively, of the 242 urine samples analyzed for NNAL, we used Friedman’s nonparametric chi-square test (Friedman 1937) as a sensitivity analysis treating preexposure, postexposure, and next-day urinary NNAL values as repeated measures on participants. We used SAS software (version 9.1; SAS Institute, Inc. Cary, NC) to perform the analyses, and all statistical tests were considered significant at α = 0.05.

## Results

We recruited 28 participants for the study (18 females). Of these, 17 were white (11 female), 7 were black (3 females), 3 were Asian (all female), and 1 described her race as other. All participants were 21–37 years of age. We excluded one participant who had a baseline (preexposure before first site visit) salivary cotinine concentration of 5.25 ng/mL—a concentration characteristic of very high SHS exposure or occasional smoking. This individual lived with a smoker. Otherwise, the range of preexposure salivary cotinine concentrations in samples provided before the first study visit confirmed that prestudy SHS exposures were low (0.011–0.480 ng/mL).

**Figure 1:**
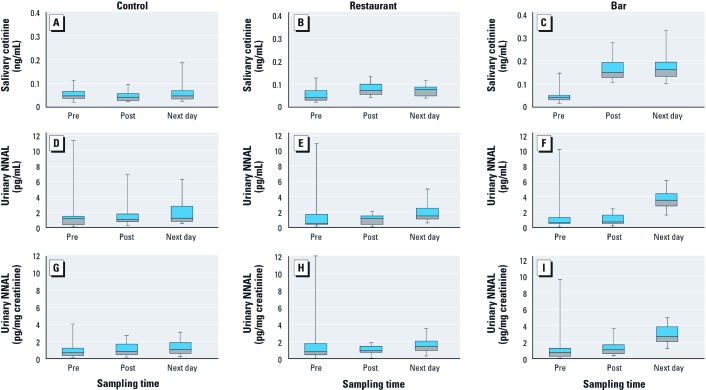
Salivary cotinine (ng/mL) for control, restaurant, and bar (*A, B, *and* C*, respectively), urinary uncorrected NNAL (pg/mL) for control, restaurant, and bar (*D–F*), and creatinine-corrected NNAL (pg/mg creatinine) for control, restaurant, and bar (*G–I*) measured preexposure, immediately after 3-hr SHS exposure, and in first-void next-day samples of participants (*n* = 27). Blue boxes represent third quartiles, black lines in the boxes are medians, gray boxes are first quartiles, and whiskers represent 5th and 95th percentiles.

*Biomarker levels according to sample and site.* One participant did not provide next-day saliva and urine samples. Also, 11 of the 242 samples collected did not have enough saliva for cotinine analysis (*n* = 1 preexposure, *n* = 7 postexposure, and *n* = 3 next-day) and 1 sample was below the LOD (0.015 ng/mL). Geometric means of preexposure, postexposure, and next-day salivary cotinine levels are presented in Table 2; quartiles and 95th percentiles of salivary cotinine for these sampling time points are presented in Figure 1A–C. Although salivary cotinine levels remained flat after participants visited the control site, salivary cotinine levels increased, as expected, after restaurant and bar visits, with greater increases after bar visits. Salivary cotinine concentrations in postexposure and next-day samples were similar. One participant’s postexposure – preexposure change in salivary cotinine reached 0.4 ng/mL after the bar visit.

For the 27 participants at baseline, NNAL was measured above the LOD in 9 (33.3%), below the LOD in 10 (37.0%), and not detected in 8 (29.6%). Overall, of 242 urine samples collected during the study period from 27 participants (81 preexposure, 81 postexposure, and 80 next-day samples, 1 did not provide a next-day urine sample), urinary NNAL was measured above the LOD in 56.2% (*n* = 136), below the LOD in 14.9% (*n* = 36), and not detected in 28.9% (*n* = 70). Geometric means for uncorrected and creatinine-corrected preexposure, postexposure, and next-day urinary NNAL are presented in Table 2, and quartiles and 95th percentiles are illustrated in Figure 1D–I. Urinary NNAL concentrations remained flat after visits to the control site. There was also a lack of change between previsit and postvisit restaurant and bar samples, respectively, in contrast to changes noted for next-day restaurant and bar samples. As expected, larger changes in urinary NNAL levels were observed in next-day samples after visits to the bar site.

*Differences in biomarker responses between sites.* Regression models to test differences in biomarkers after exposure between sites excluded two participants whose samples were not collected according to their preassigned sequence because of personal scheduling conflicts, and one participant who did not complete all three visits. As a result, we had eight complete Latin squares with three participants per square (24 participants total).

**Figure 2:**
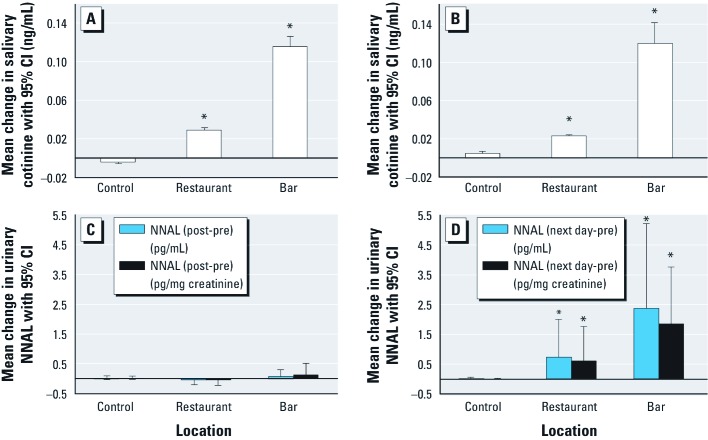
Saliva-cotinine and urinary-NNAL changes after a 3-hr visit to outdoor seating areas of a restaurant and a bar where outdoor smoking was allowed and to an open-air control location with no smokers. (*A*) Salivary cotinine postexposure minus preexposure (ng/mL). (*B*) Salivary cotinine next-day minus preexposure (ng/mL). Urinary uncorrected (pg/mL) and creatinine-corrected postexposure minus preexposure NNAL (pg/mg creatinine) (*C*), and urinary uncorrected (pg/mL) and creatinine-corrected (pg/mg creatinine) next-day minus preexposure NNAL (*D*). Values represent differences in geometric means and 95% CIs. *Statistically higher than control at α = 0.05 level of significance.

Mean differences in postexposure versus preexposure salivary cotinine concentrations were significantly greater after visits to the bar location [0.115 ng/mL (95% CI: 0.105, 0.126)] and restaurant location [0.030 ng/mL (95% CI: 0.028, 0.031)] relative to changes observed after visiting the control site [–0.004 ng/mL (95% CI –0.005, –0.003)] (Figure 2A), with *p* < 0.001 for both comparisons based on regression models (Table 3). We obtained similar results when we looked at next-day versus preexposure concentrations (Table 3, Figure 2B).

**Table 3:** Test of effect of exposure location and pair-wise comparisons between location types.

Analyte	Response variable	Fixed effect and comparisons		Estimatea	SE	F or t-valueb	p-Value
Cotinine		log(post) – log(pre)		Fixed effect	Location						76.72		< 0.001
				Comparisonsc	Restaurant vs. control		0.264		0.049		5.32		< 0.001
					Bar vs. control		0.596		0.048		12.36		< 0.001
					Bar vs. restaurant		0.334		0.049		6.72		< 0.001
NNAL				Fixed effect	Location						1.62		0.210
C-C NNAL				Fixed effect	Location						2.05		0.142
Cotinine		log(next-day) – log(pre)		Fixed effect	Location						40.99		< 0.001
				Comparisonsc	Restaurant vs. control		0.180		0.065		2.75		0.008
					Bar vs. control		0.570		0.064		8.83		< 0.001
					Bar vs. restaurant		0.390		0.065		6.05		< 0.001
NNAL				Fixed effect	Location						6.30		0.004
				Comparisonsc	Restaurant vs. control		1.458		0.561		2.60		0.012
					Bar vs. control		1.915		0.561		3.42		< 0.001
					Bar vs. restaurant		0.456		0.548		0.83		0.411
C-C NNAL				Fixed effect	Location						6.16		0.005
				Comparisonsc	Restaurant vs. control		1.370		0.552		2.48		0.001
					Bar vs. control		1.884		0.552		3.41		< 0.001
					Bar vs. restaurant		0.553		0.541		0.95		0.348
C-C, creatinine corrected. aDifference in least square means on log10 scale (when back-transformed, represents a ratio of concentrations of biomarkers between sites). bF-value applies to fixed effects test, whereas t-values apply to pair-wise comparisons; pair-wise comparisons were not made when overall F tests were nonsignificant; cp-Values adjusted for multiple comparisons (Tukey’s method).													

As noted above, there were no significant differences in urinary (uncorrected and creatinine-corrected) NNAL concentrations for samples collected immediately after site visits compared with previsit samples, regardless of site (Figure 1D-I). Consistent with expectations, there also were no significant differences in changes observed between sites (Figure 2C). However, contrasts between next-day and previsit uncorrected and creatinine-corrected urinary NNAL concentrations were significantly greater after visits to the bar location (*p* < 0.001) and restaurant location (*p* = 0.006) compared with the control site (Table 3, Figure 2D). Further, when we analyzed preexposure, postexposure, and next-day NNAL as repeated measures (Friedman's nonparametric chi-square test), location type had a significant effect on the distribution of urinary NNAL (uncorrected: χ^2^ = 7.16, *p* = 0.028; creatinine corrected: χ^2^ = 13.9, *p* = 0.001). These results are consistent with those presented above.

*Differences in weekly preexposure biomarkers (crossover effect)*. Compared with baseline average salivary cotinine levels [0.038 ng/mL (95% CI: 0.029, 0.049)], week-3 preexposure salivary cotinine was significantly higher [0.055 ng/mL (95% CI: 0.043, 0.071); *p* = 0.006]. Average week-2 preexposure salivary cotinine levels [0.045 ng/mL (95% CI: 0.035, 0.058)] were not significantly different from baseline (*p* = 0.416). Preexposure urinary NNAL did not differ significantly during the 3 weeks of the study (uncorrected, *p* = 0.778; creatinine corrected, *p* = 0.169). Weeks 1, 2, and 3 geometric means and 95% CI for preexposure creatinine-corrected NNAL were as follows: 0.5 (95% CI: 0.3, 0.7), 0.8 (95% CI: 0.5, 1.2), and 0.7 (95% CI: 0.5, 1.1) pg/mg creatinine, respectively.

*Biomarkers versus cigarette counts.* On average, we counted a higher number of lit cigarettes over the 3-hr sampling period outside the bar (mean ± SD, 144.5 ± 39.9) than outside the restaurant (mean ± SD, 33.5 ± 28.0). No lit cigarettes were observed at the control site. The effect of cigarette count on postexposure minus preexposure changes in salivary cotinine [0.0032 (0.0003), presented as effect estimate and SE] and on next-day minus preexposure changes in salivary cotinine [0.0032 (0.0004)] were significant (both *p* <0.001). Just as with location type, cigarette count was not associated with postexposure minus preexposure changes in urinary uncorrected and creatinine-corrected NNAL but was significantly associated with next-day minus preexposure changes in uncorrected NNAL [0.0003 (0.0007)] and creatinine-corrected NNAL [0.0032 (0.0008)] (both *p* = 0.018).

*Salivary cotinine versus urinary NNAL correlations and ratios.* Changes in salivary cotinine were generally moderately correlated to changes in urinary NNAL (uncorrected and creatinine corrected); higher correlations were observed between changes in postexposure – preexposure and next-day – preexposure salivary cotinine and in next-day – preexposure changes in NNAL (Table 4). Although the ratios of urinary NNAL to salivary cotinine did not differ significantly across location type, ratios were lower in postexposure samples compared to preexposure and next-day samples [see Supplemental Material, Table 2 (http://dx.doi.org/10.1289/ehp.1104413)].

**Table 4:** Spearman rank correlation coefficients between changes measured in salivary cotinine and urinary NNAL.

Changes between sampling times	Cotinine Post – Pre	Cotinine Next day – Pre	NNAL Post – Pre	NNAL Next day – Pre	C-C NNAL Post – Pre	C-C NNAL Next day –Pre
Cotinine (post – pre)		1 (75)		0.78* (73)		0.12 (75)		0.42* (74)		0.21 (75)		0.48* (74)
Cotinine (next day – pre)				1 (76)		0.22 (76)		0.49* (76)		0.33* (76)		0.60* (76)
NNAL (post – pre)						1 (81)		0.47* (80)		0.75* (81)		0.36* (80)
NNAL (next day – pre)								1 (80)		0.46* (80)		0.80* (80)
C-C NNAL (post – pre)										1 (81)		0.53* (80)
C-C NNAL (next day – pre)												1 (80)
C-C, creatinine-corrected urinary NNAL concentrations; next-day–pre, next-day minus preexposure; post–pre, postexposure minus preexposure. Number of observations are shown in parentheses. *p < 0.001.												

## Discussion

In this study, we investigated the uptake of tobacco-specific compounds in 27 nonsmokers after they were exposed to SHS outside a restaurant and a bar in Athens, Georgia. We observed significant increases in cotinine measured in saliva collected both immediately after and the morning after the 3-hr visits outside bar and restaurant sites, and observed significant increases in urinary NNAL (uncorrected and creatinine corrected) measured in urine collected at first-morning void after bar and restaurant site visits. In contrast, there were minimal changes in biomarker levels after 3-hr visits to a control location without smokers present. The changes in salivary cotinine and urinary NNAL measured after visits to bar and restaurant locations were significantly greater than were changes after control site visits. In addition, we observed significant associations between the number of cigarettes smoked and changes in biomarker levels after visits.

Cotinine has been proposed as a very sensitive and specific biological marker of SHS exposure (Benowitz 1999). Cotinine has an average half-life of 16 hr and is eliminated from the body within 3–4 days after the last exposure (Benowitz 1996). Salivary cotinine was used in the current study instead of the commonly used serum cotinine because it is easier to collect. Previous studies have shown that serum and salivary cotinine levels are highly correlated with ratios ranging from 1.1 to 1.4 (Curvall et al. 1990). We previously used salivary cotinine to characterize uptake of SHS constituents in nonsmokers after a 6-hr visit to outdoor patios and seating areas of restaurants and bars (Hall et al. 2009). Geometric mean changes in salivary cotinine after visits to outdoor patios of bars and restaurants were 0.114 ng/mL and 0.039 ng/mL, respectively. These changes were significantly higher than those observed after visits to a control site (0.006 ng/mL) (Hall et al. 2009) and were similar to what we report in the current study, despite longer exposure times in the first study (6 hr compared with 3 hr). However, this finding is consistent with expectations given that visits for the present study were timed to occur when smoking activity was highest at each site (1800–2100 hours and 2300–0200 hours) at the restaurant and bar sites, respectively).

To our knowledge, we are the first study to report changes in urinary NNAL concentrations following outdoor SHS exposure. NNAL is the metabolite of NNK. Both are systemic pulmonary carcinogens specific to tobacco smoke (Hecht 2003). There is strong evidence that NNK is a causative agent in the formation of lung adenocarcinoma in smokers (Hoffmann et al. 1996), which is now the most frequent type of lung tumor found in nonsmokers (Hoffmann et al. 1996). Total NNAL has been used to characterize human exposure to carcinogenic tobacco-specific nitrosamines among nonsmokers with exposure to SHS (Anderson et al. 2001). In addition, epidemiologic studies linking lung cancer to SHS have been strengthened by the detection of NNAL in nonsmokers (DHHS 2006).

We observed significant increases in total uncorrected and creatinine-corrected NNAL measured in next-day first-void urine samples following both bar and restaurant site visits compared to the control site visit. The lack of changes in urinary NNAL immediately following exposure are consistent with elimination half-life of NNAL, which averages 10–16 days (Goniewicz et al. 2009). Although NNAL concentrations measured in the current study were low, NNAL was detected above the LOD in 66 of the 80 next-day samples (83%) compared with 38 of the 81 (47%) postexposure and 32 of 81 (40%) preexposure urine samples. Our results demonstrate that nonsmokers with even brief SHS exposures (3 hr) may be exposed to detectable levels of tobacco-specific nitrosamines.

We observed significant but moderate correlations between salivary cotinine and next-day urinary NNAL showing that salivary cotinine from outdoor SHS exposure may not always be highly predictive of urinary NNAL concentrations. This may reflect differences in the metabolic rates of these compounds, as well as differences in internal doses received after exposure to the parent compounds in the ambient atmosphere. Unpublished research from the Philip Morris Tobacco Company showed that NNK was formed in sidestream smoke in a chamber and in indoor air hours after it was released (Schick and Glantz 2007) whereas air–nicotine concentrations decreased over time. Thus, as SHS ages, exposure to NNK can increase, while nicotine decreases. The extent to which this applies in outdoor locations is uncertain and needs further study. We also present novel data on the ratios between urinary NNAL and salivary cotinine in Supplemental Material (http://dx.doi.org/10.1289/ehp.1104413).Although we did not see differences across locations, our results seem to indicate that the ratio of urinary NNAL to salivary cotinine is lower immediately following SHS exposure compared with before exposure and next-day. This seems to suggest that the NNAL to cotinine ratio may indicate time from last exposure, with low ratios signaling recent SHS exposures. Further investigation is needed to assess its applicability.

Studies of indoor exposure to SHS among nonsmokers have previously reported changes in urinary NNAL ranging from 3.8 pg/mg creatinine to 12.3 pg/mL (Anderson et al. 2003; Parsons et al. 1998), at least twice the next-day – preexposure changes in NNAL after the bar site visits reported here. However, by measuring NNAL in first-morning void samples, it is likely that we underestimated NNAL changes after the bar visits relative to restaurant visits (i.e., shorter time from postexposure to next-day samples after bar visits compared to the restaurant visits). Also, the NNAL changes following bar visits we report here are at least two orders of magnitude lower than what has been measured in active smokers (Stepanov and Hecht 2005). Although the clinical significance of outdoor SHS levels we present here are not fully known, asthmatic adults with low level outdoor SHS exposure measured using a nicotine badge (nicotine 0.03 μg/m^3^) showed increased risk of respiratory symptoms and extra bronchodilator use (Eisner et al. 2001). Personal air nicotine reported by Eisner et al. is not easily comparable with systemic cotinine and NNAL doses reported here. Studies have also shown significant cardiovascular effects of low level SHS exposure (Barnoya and Glantz 2005). Repace et al. (1998) estimated an increased lifetime mortality risk of 1 per 1,000 persons for lung cancer and 1 per 100 persons for heart disease from an average salivary cotinine of 0.4 ng/mL. Although average salivary cotinine levels from SHS exposure outside the bar were 25% of this estimated salivary cotinine level, one participant had an increase of 0.4 ng/mL. Given that urinary and possibly salivary cotinine underestimate NNK exposure (Benowitz et al. 2010) and therefore cancer risk estimates, the NNAL levels reported here will help improve these disease risk estimates. The reported NNAL concentrations also raise concerns about hospitality workers who are potentially exposed to outdoor SHS for longer periods and more frequently during the week than the study participants.

The present study has several strengths and limitations. Among the strengths, we used a crossover design that controls for the high inter- and intraindividual variability commonly observed in biomarker response. Further, this study is the first to report NNK exposure by measuring NNAL in nonsmokers exposed to outdoor SHS—results that could potentially have public health implications. On the other hand, one of the limitations of the study is the absence of objective proxies of SHS levels such as PM_2.5_, CO, or air nicotine. Instead we used cigarette count to assess SHS at each site, which showed an exposure response with biomarkers. Further, we did not collect variables such as temperature and wind speed at the study sites that would more accurately characterize exposure because the study sites were different. Our results may not be generalizable to other sites of outdoor SHS exposure given that the extent of exposure will vary depending on the amount of smoking, ventilation, wind speed, and other factors. However, contrasts between the three sites do show differences according to moderate and high exposures (at the restaurant and bar, respectively).

Finally, there is a possibility of carryover effect from week to week in biomarker response. No significant carryover effect was observed for NNAL, but we observed higher preexposure salivary cotinine in week 3 compared with baseline. It is possible that some participants had short unreported exposures to SHS before study times in week 3. Nevertheless, as we investigated the changes between sampling time points, these response variables are less likely to be affected by carryover effects.

## Conclusions

Our results indicate that both salivary cotinine and urinary NNAL increased significantly following exposure to SHS outside of restaurants and bars in Athens, Georgia. Although urinary total NNAL concentrations were relatively low, they indicate that nonsmokers exposed to brief periods of SHS in outdoor locations may be exposed to measurable concentrations of carcinogenic tobacco-specific nitrosamines such as NNK. Because of the relatively long half-life of NNAL and much lower NNK exposure in passive smokers than in active smokers, future studies that investigate outdoor SHS exposure should consider measuring NNAL in urine of nonsmokers at ≥ 24 hr postexposure.

## Supplemental Material

(57 KB) PDF
