# Supplementary material for: Exposure to Secondhand Smoke Outside of a Bar and a Restaurant and Tobacco Exposure Biomarkers in Nonsmokers
Source: Environ Health Perspect. 2012 Apr 6;120(7):1010–6. doi: 10.1289/ehp.1104413 (PMC3404659; doi:10.1289/ehp.1104413)
Supplement: (57 KB) PDF [file ehp.1104413.s001.pdf]

SUPPLEMENTAL MATERIAL

Exposure to Secondhand Smoke Outside of a Bar and a Restaurant and Tobacco Exposure Biomarkers in Non-smokers

Gideon St.Helen, J. Thomas Bernert, Daniel B. Hall, Connie S. Sosnoff, Yang Xia, John R. Balmes, John E. Vena, Jia-Sheng Wang, Nina T. Holland, Luke P. Naeher

Table of Contents

Supplemental Material, Methods 2

Supplemental Material, Results 2

Supplemental Material, Discussion 3

Supplemental Material, References 3

Supplemental Material, Table 1: Illustration of replicated Latin square 4

Supplemental Material, Table 2: Urinary NNAL to salivary cotinine ratio among study participants 6

Supplemental Material, Methods

The ratio urinary NNAL to urinary cotinine has been shown to be much higher in passive smokers compared to active smokers (Benowitz et al. 2010). There are currently no studies reporting the ratio of NNAL to cotinine in non-smokers exposed to outdoor SHS. Thus we computed geometric means of the ratios between uncorrected urinary NNAL and salivary cotinine as well as creatinine-corrected NNAL and salivary cotinine and we used non-parametric Kruskal-Wallis analysis of variance to compare these ratios by exposure location.

Supplemental Material, Results

Geometric means and 95% CI for the ratios between urinary NNAL measured in pg/mL (either uncorrected or corrected to urinary creatinine concentration in pg/mL) and salivary cotinine measured in ng/mL are given in Supplemental Material, Table 2. Ratios computed are for biomarker levels at pre-exposure, post-exposure, and next-day sampling times, respectively. There was no significant difference in pre-exposure urinary NNAL:salivary cotinine ratios across location type (uncorrected and creatinine-corrected NNAL). A marginally non-significant difference in post-exposure ratios (uncorrected NNAL) was observed by location-type (χ^2 = 5.76, p = 0.056), with lower ratios following visits to the restaurant and bar sites compared to the control site. This was non-significant when corrected for creatinine (χ^2 = 4.29, p = 0.117). Location differences were not observed when next day ratios were considered.

Supplemental Material, Discussion

We present the first set of data on urinary NNAL:salivary cotinine ratios following outdoor SHS exposure among non-smokers. We did not observe significant differences in pre-

exposure and next-day NNAL:cotinine ratios, respectively, across study location. However, our data seem to suggest that post-exposure NNAL:cotinine ratios were marginally lower following visits to bar and restaurant sites compared to the control location. Supplemental Material, Table 2 also shows a clear trend in the ratios following SHS exposure, with low ratios immediately post-exposure and higher ratios at pre-exposure and next-day time points. The low post-exposure ratios indicate significant increases in salivary cotinine immediately following end of SHS exposure while NNAL concentrations remained unchanged. It seems low versus high NNAL:cotinine ratios in passive smokers would indicate time from most recent exposure, where low ratios would be indicative of more recent SHS exposure. This warrants further investigation as a tool in epidemiologic studies to characterize passive smokers by time from most recent SHS exposure.

Supplemental Material, References

Benowitz NL, Goniewicz M, Eisner M, Lazcano-Ponce E, Zielinska-Danch W, Koszowski B, et al. 2010. Urine cotinine underestimates exposure to tobacco-derived lung carcinogen 4-(methylnitrosamino)-1-(3-pyridyl)-1-butanone (NNK) in passive compared to active smokers. Cancer Epidemiology Biomarkers & Prevention 19(11): 2795-2800.

Supplemental Material, Table 1 Illustration of replicated Latin square design used in the study

Day/Week	Subject					Number				
Friday (F)	F1	F2	F3	F4	F5	F6	…	Fn-2	Fn-1	F12
1	A	B	C	A	B	C		A	B	C
2	B	C	A	C	A	B		B	C	A
3	C	A	B	B	C	A		C	A	B
Saturday (S)	S13	S14	S15	S16	S17	S18	…	Sn-2	Sn-1	S24
1	A	B	C	A	B	C		A	B	C
2	B	C	A	C	A	B		B	C	A
3	C	A	B	B	C	A		C	A	B

Each box represents one Latin square. The three exposure sites, bar, restaurant, and control, are depicted by letters A, B, C, which are arranged in 3 x 3 Latin squares to accommodate a minimum of 24 subjects (F1-F12 and S13 to S24) on whom data were collected over three (3) sampling days (Friday or Saturday evenings for a given subject)

Supplemental Material, Table 2 Urinary NNAL to salivary cotinine ratio among study participants by study location and gender

Group		†NNAL to Cotinine Ratio			Creatinine-corrected †NNAL to Cotinine Ratio		
		**Pre-exposure**	**Post-exposure**	**Next day**	**Pre-exposure**	**Post-exposure**	**Next day**
Control	n	17	17	16	17	17	16
	GM	19.6	21.3	23.1	15.7	17.9	16.2
	95% CI	(10.8, 35.6)	(13.5, 33.5)	(13.8, 38.6)	(9.5, 25.8)	(12.9, 24.8)	(10.1, 25.9)
	Range	2.3, 148.7	2.1, 103.0	1.7, 91.3	4.0, 79.6	5.0, 40.7	2.0, 51.6
Restaurant	n	18	11	23	18	11	23
	GM	15.6	10.3	21.2	15.3	12.0	19.2
	95% CI	(8.3, 29.5)	(6.1, 17.6)	(15.6, 28.8)	(8.6, 27.2)	(7.7, 18.7)	(13.5, 27.3)
	Range	2.4, 320.6	1.7, 25.0	6.2, 88.0	2.2, 466.9	2.3, 25.6	4.4, 126.5
Bar	n	18	19	25	18	19	25
	GM	13.4	5.0	21.1	14.2	6.9	16.1
	95% CI	(7.7, 23.2)	(3.2, 7.8)	(16.3, 27.2)	(7.1, 28.5)	(4.7, 10.2)	(12.5, 20.8)
	Range	1.7, 141.7	1.0, 19.0	3.6, 102.9	2.9, 1340.3	1.3, 26.4	5.8, 59.9
Females	n	31	32	42	31	32	42
	GM	18.5	11.1	20.7	18.0	11.4	18.1
	95% CI	(11.9, 28.7)	(7.8, 15.8)	(16.3, 26.3)	(12.3, 26.2)	(8.5, 15.3)	(14.2, 23.1)
	Range	1.7, 320.6	1.2, 50.0	1.7, 88.0	2.9, 466.9	2.3, 34.7	2.0, 126.5
Males	n	22	15	22	22	15	22
	GM	12.9	8.0	23.4	11.7	10.5	15.6
	95% CI	(7.8, 21.3)	(4.0, 15.9)	(17.3, 31.6)	(6.5, 21.1)	(6.5, 16.8)	(11.4, 21.3)
	Range	2.4, 148.7	1.0, 103.0	8.0, 102.9	2.2, 1340.3	1.3, 40.7	4.2, 59.9
All Subjects	n	53	47	64	53	47	64
	GM	15.9	10.0	21.6	15.0	11.1	17.2
	95% CI	(11.5, 22.0)	(7.3, 13.7)	(18.0, 26.0)	(10.9, 20.8)	(8.7, 14.1)	(14.2, 20.8)
	Range	1.7, 320.6	1.0, 103.0	1.7, 102.9	2.2, 1340.3	1.3, 40.7	2.0, 126.5

Note: GM = geometric mean; CI = confidence interval; †NNAL concentration measured in pg/ml or pg/mg creatinine and cotinine in ng/mL; NNAL = 4-(methylnitrosamino)-1-(3-pyridyl)-1-butanol
